# Supplementary material for: Theoretical insights into the Peierls plasticity in SrTiO3 ceramics via dislocation remodelling
Source: Nat Commun. 2022 Nov 14;13:6925. doi: 10.1038/s41467-022-34741-4 (PMC9663548; doi:10.1038/s41467-022-34741-4)
Supplement: Supplementary file 1 — Supplementary Information [file 41467_2022_34741_MOESM1_ESM.pdf]

Supplementary Information

Theoretical insights into the Peierls plasticity in $SrTiO_3$ ceramics via dislocation remodelling

Yi Li et.al

Content

1. Supplementary Methods 1

1.1 Molecular Dynamics (MD) simulations on the [011]$(0\bar{1}1)$ edge dislocation in $SrTiO_3$ 1

1.2 Sample preparation 1

2.Supplementary Discussions 2

Supplementary references 11

1. Supplementary Methods

1.1 Molecular Dynamics (MD) simulations on the [011]$(0\bar{1}1)$ edge dislocation in $SrTiO_3$

The properties of [011]$(0\bar{1}1)$ edge dislocation of $SrTiO_3$ were simulated based on molecular dynamics by using the LAMMPS code. The atomic interaction potentials developed by Thomas et al. [1] were applied in this paper. The parameters of the potential were set according to the report of Yildiz et al. [2]. A 10×64×64 supercell (~201 550 atoms) was applied to simulate the $SrTiO_3$ bulk. All the calculations applied long-range Coulombic interactions using the PPPM method. The cutoff distance of short-range interaction was set as 12 Å. The simulations of dynamic processes are performed at 300 K in the NVT ensemble.

1.2 Sample preparation

The samples for the compression tests were prepared by the following processes. The quadrangular prisms with dimensions 2×2×4 mm^3 were cut from the initial

materials. Then, the prisms were polished and no slip traces were observed on the surfaces. The polished samples were divided into two groups. One is reduced at 1450 ºC for 6 hours in Argon atmosphere as the oxygen-deficient samples, and the other is also heated at 1450 ºC for 6 hours in air atmosphere as the reference samples

2.Supplementary Discussions

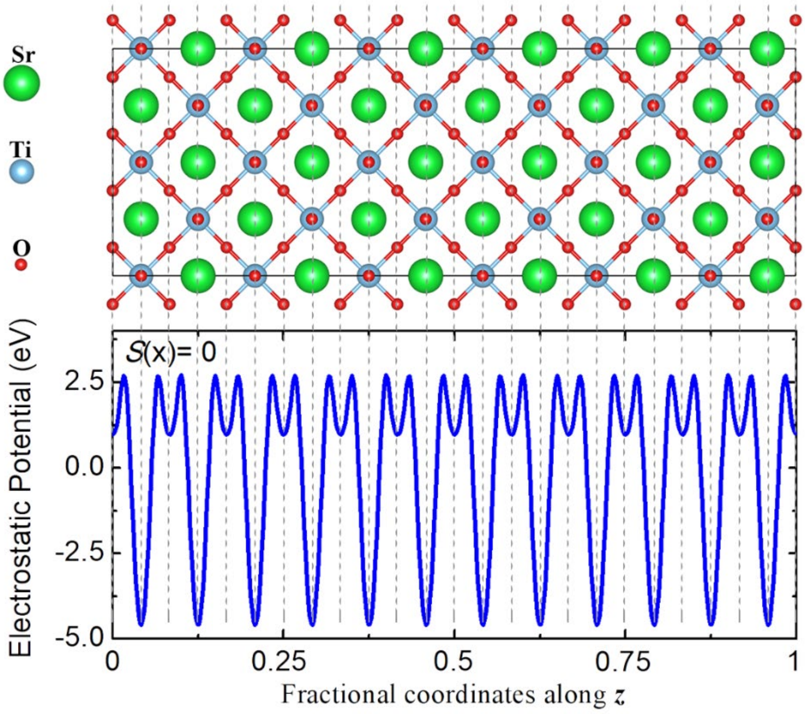

Supplementary Figure 1 Planar averaged electrostatic potentials along $[0\bar{1}1]$ direction for the supercell without shear. The plot has valleys and peaks. The low and high valleys correspond to the SrTiO and O_2 layers, respectively.

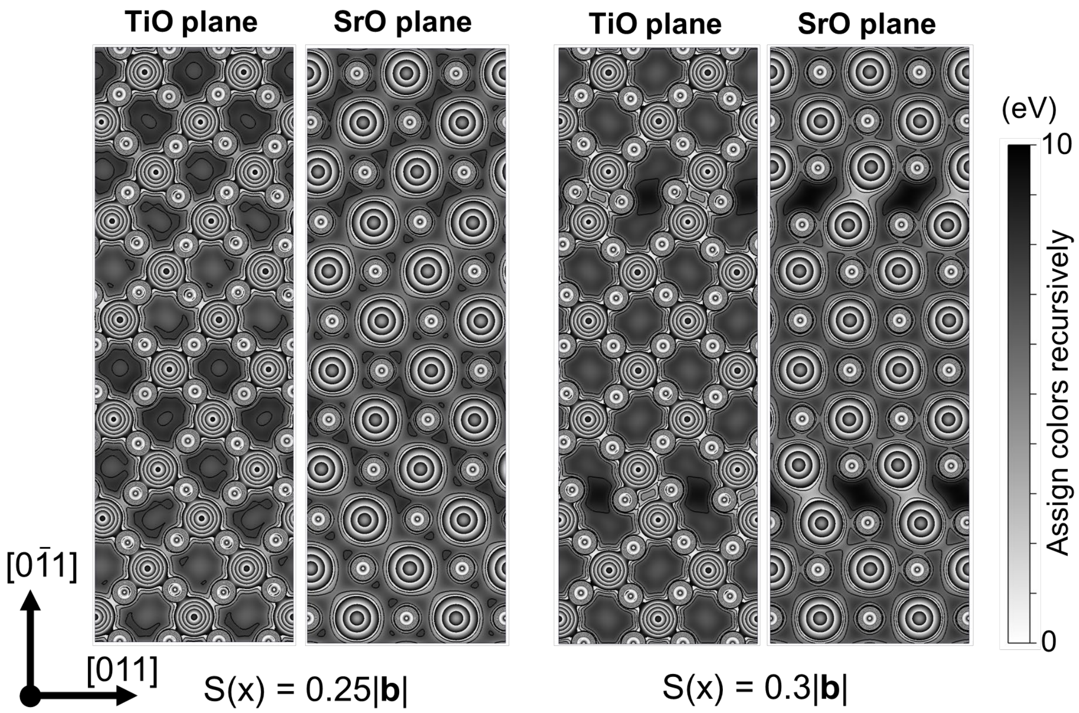

Supplementary Figure 2 2D displays of the electrostatic potentials on (100) Ti-O and Sr-O planes for the supercell with the shear displacements of 0.25|***b***| and 0.3|***b***|.

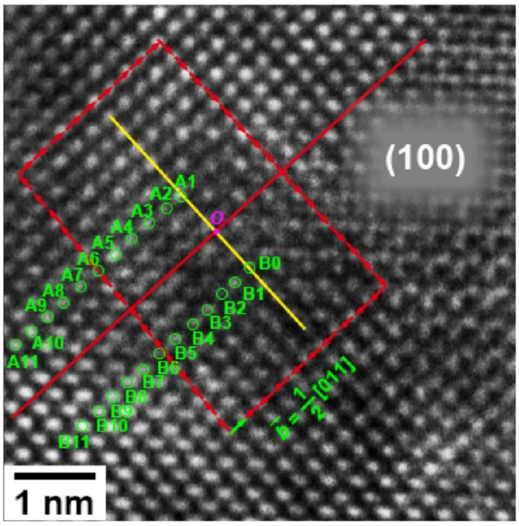

Supplementary Figure 3 high-resolution transmission electron microscopy (HRTEM) image of $SrTiO_3$ crystal from a [100] perspective. HRTEM image containing a half <011>{011}-type dislocation core in [100] oriented $SrTiO_3$ crystal. The green circles mark the atomic positions and are numbered as A1, A2, …, A11, B0, B1, …, B11. The pink point *O* marks the center of the dislocation core.

The local misfit energy is calculated by applying shear displacements [S(x)] to the atoms on one side of glide plane. The values of S(x) in our model are measured through the atoms that are two SrTiO layers apart from the glide plane (Fig. 1). Therefore, the atoms selected to calculate S(x) in Supplementary Figure 3a are two atomic layers apart from the glide plane (red line). The shear displacements [S(x)] and the distance x are calculated by:

$$S_n(\mathrm{x}_n)=\frac{\overrightarrow{\mathrm{B}_n\mathrm{A}_n}\cdot\vec{\delta}}{|\vec{\delta}|} \qquad (1),$$

$$\mathrm{x}_n=\frac{\overrightarrow{\mathrm{B}_n O}\cdot\vec{\delta}}{|\vec{\delta}|} \qquad (2),$$

where n is the atomic number 1, 2, …, 11 and $\vec{\delta}$ is a vector that is parallel to the red line which marks the glide plane in Supplementary Figure 3a.

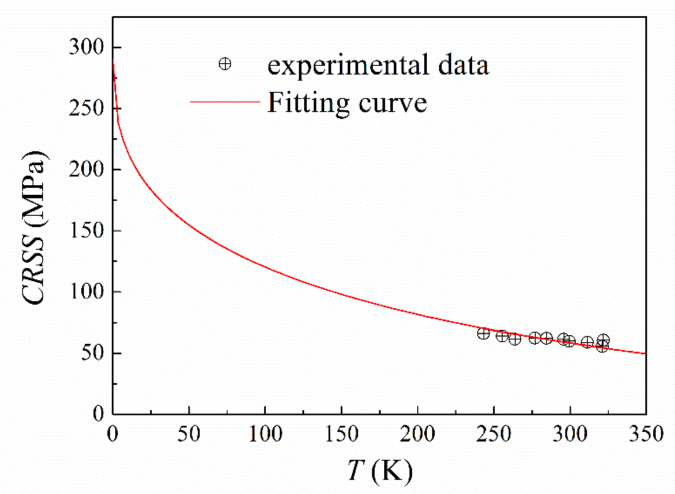

Supplementary Figure 4 Extrapolation of CRSS calculated by fitting the experimental data that corresponds to <110>{110} edge dislocation [3].

In order to compare the calculated Peierls stress σ_{PN} with the experimentally measured critical resolved shear stress (CRSS), an extrapolation up to the measured temperatures has been performed following the reference reported by P. Carrez et al. [4]:

$$\Delta H^*(\sigma) = \Delta H_0[1-(\sigma/\sigma_{PN})^p]^q$$

where ΔH^* is the critical enthalpy of the configuration as a function of the effective stress σ, p and q are the fitting parameters. The values of ΔH_0= 2.4 eV, p= 0.5 and q= 2.5 are applied according to P. Carrez's work. $\Delta H^*(\sigma)$ can be converted into a function of critical resolved shear stress at constant strain rate and follows an Arrhenius-type relationship: $\Delta H^*(\sigma) = Ck_BT$, where k_B is the Boltzmann constant, T is the absolute temperature and C is a factor that depends on σ and plastic strain rate. In the standard experimental conditions, C is usually in the range of 20-30 [5, 6], and a value of 21 is applied. An extrapolation of CRSS has been performed to 0 Kelvin based on the experimental data [3]. As shown in Supplementary Figure 4, the extrapolated CRSS is 290 MPa at 0 Kelvin, with which the calculated Peierls stress (305 MPa) in our manuscript agrees well.

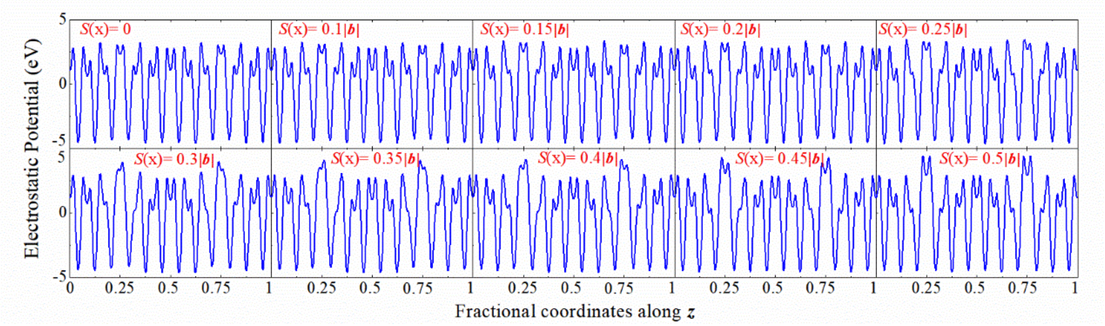

Supplementary Figure 5 Planar averaged electrostatic potentials along ***z*** direction, corresponding to $[0\bar{1}1]$, for the oxygen-deficient supercell with the shear displacements S(x) of 0, 0.1|***b***|, 0.15|***b***|, 0.2|***b***|, 0.25|***b***|, 0.3|***b***|, 0.35|***b***|, 0.4|***b***|, 0.45|***b***| and 0.5|***b***|.

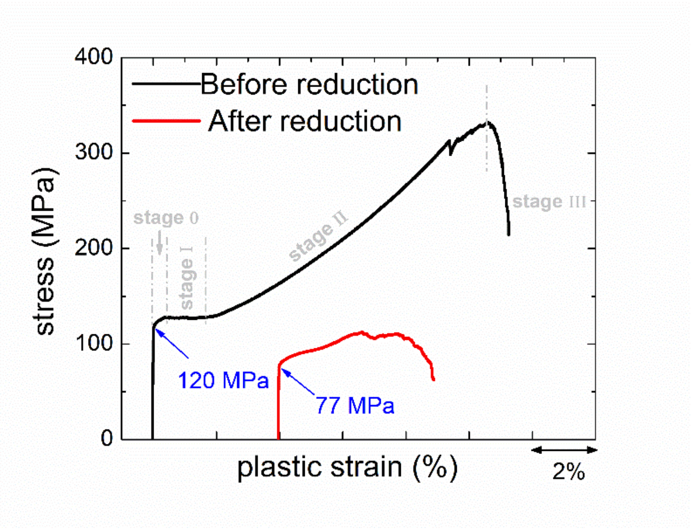

Supplementary Figure 6 Stress-plastic strain curves of the {100} $SrTiO_3$ crystals before and after oxygen reduction.

The yield stress corresponds to the beginning of plastic deformation. According to Yang et al.'s work [7], the plastic strain of un-reduced specimen can be divided into four stages: 0, I, II and III, which are described by the mechanisms of axis rotation, easy glide, multiple slip forming the cell-and-wall structure, and work softening, respectively. The stage II corresponds to the multiplication process of dislocations. The dislocation density increases up to the maximum at the end of stage II, and then decreases because of the annihilation of dislocations in stage III. After reduction, the strain at the end of stage II decreases, indicating that oxygen vacancies facilitate the multiplication process of dislocations. The result supports our simulations that oxygen vacancies contribute to the dislocation nucleation.

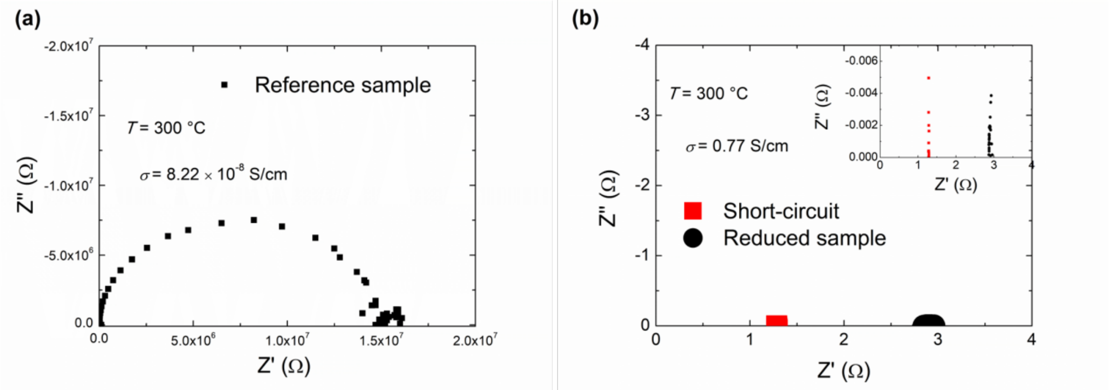

Supplementary Figure 7 Nyquist plot of impedance for (**a**) the reference sample (without reduction treatment) and (**b**) the reduced sample at 300 ºC. The red squares refer to the measured data for the short-circuited sample. The insert figure of Fig. S7(b) shows the zoom-in on the scale of Z".

The impedance spectroscopy measurements are applied to compare the electrical conductivity of the reference sample (without reduction treatment) and the reduced sample, which can be regarded as a criterion on the oxygen vacancy concentration in $SrTiO_3$ [8]. Oxygen vacancies and electrons are introduced simultaneously ($O_O^{\times} \rightarrow V_O^{\cdot\cdot} + 2e' + 1/2O_2$) during the reduction treatment, and they can contribute to the improving the electrical conductivity. The dominant electrical conduction behavior can be either electronic or ionic type, which depends on the temperature and oxygen vacancy concentration [9]. The reduced sample has a weak frequency response as shown in Supplementary Figure 7(b), indicating that the electronic conductivity plays the dominant role. Besides, the electrical resistance of the reduced sample is too low, so we short-circuited the sample and measured the impedance again (red squares in the figure) in order to eliminate the influence of wires and electrodes. The electrical conductivity of $SrTiO_3$ increases from 8.22 E-8 S/cm to 0.77 S/cm after the reduction treatment, indicating a high oxygen vacancy concentration in the reduced sample.

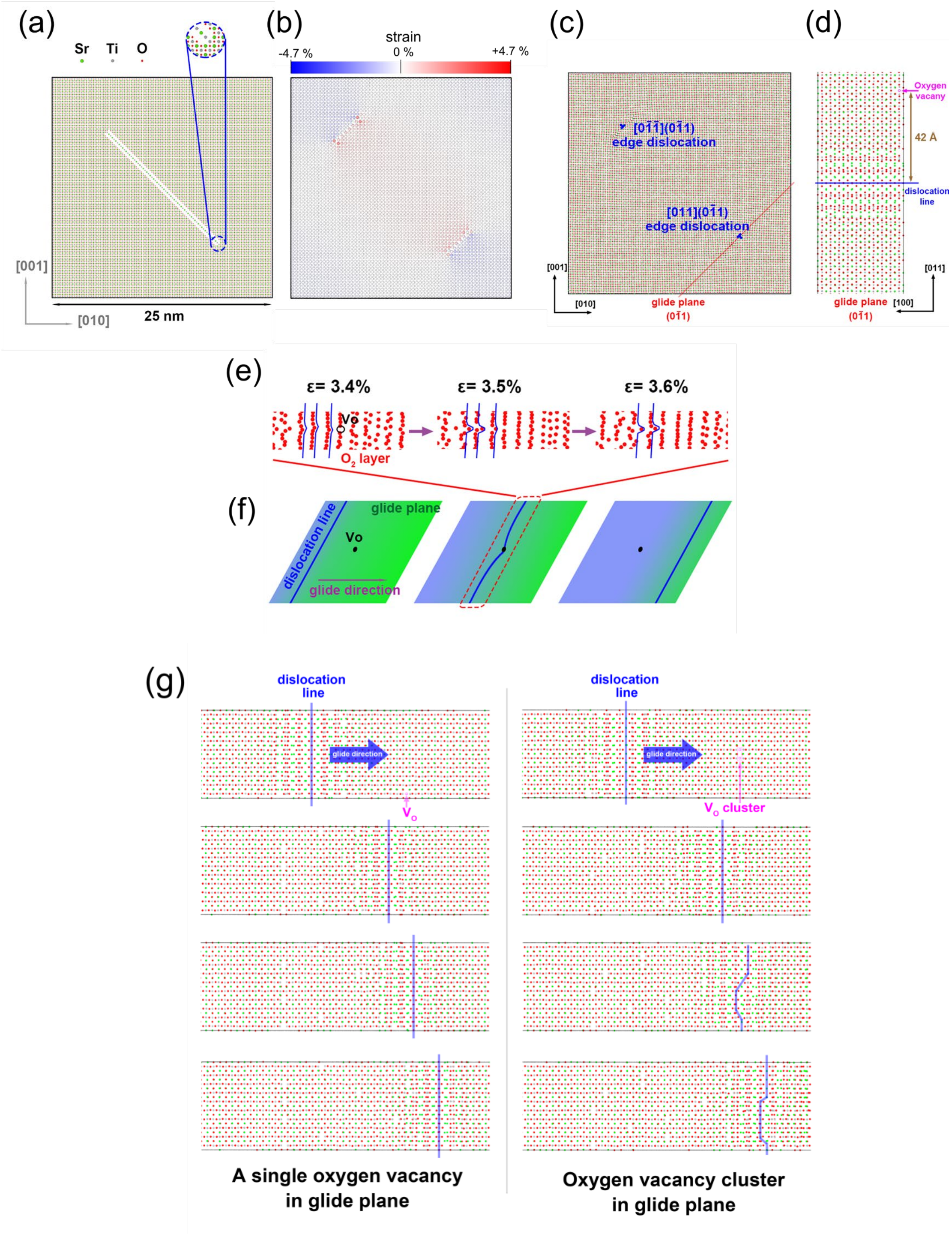

Supplementary Figure 8 Molecular dynamics simulations. (**a**) Schematic diagram for building the [011](0$\bar{1}$1) edge dislocations; (**b**) strain map for the $SrTiO_3$ box after the static relaxation; (**c**) $SrTiO_3$ structure with the two dislocations after the relaxation at 300 K; (**d**) the top view of the glide plane marked by the red line in Fig. S7c. (**e**) Atomic positions of the O_2 layer next to the glide plane in MD calculations with strain ε= 3.4%, 3.5% and 3.6%; (**f**) schematic diagram for the interaction between the [011](0$\bar{1}$1) edge dislocation and an oxygen vacancy; (**g**) the migration of dislocation

line on the glide plane with a single oxygen vacancy (left figure) and a cluster of three oxygen vacancies (right figure).

The MD simulations were applied in order to verify the effect of V_O on the glide of the $[011](0\bar{1}1)$ edge dislocation in $SrTiO_3$. As shown in Supplementary Figure 8a, the $[011](0\bar{1}1)$ edge dislocations were introduced by cutting the middle of the box along the $[0\bar{1}1]$ direction. The Burgers vector of the $[011](0\bar{1}1)$ edge dislocation passes through five crystallographic planes. The four of them corresponding to two SrTiO planes and two O_2 planes were removed, and the rest one was moved to the center of the cut as a seed of the dislocations. The cut was healed by a static relaxation under pressure, and two opposite edge dislocations were formed. Then the box was statically relaxed without pressure. Strain map for the $SrTiO_3$ box after the static relaxation is shown in Supplementary Figure 8b. It is clear that the system is large enough to prevent the interaction between the dislocations. The half-width of dislocation core is about 23 Å, which is larger than the value of 14.9 Å calculated by the PN model in the former section.

An oxygen vacancy (V_O)was introduced near the dislocation core in the glide plane. The distance between the vacancy and the center of dislocation core is about 42 Å. The structure was relaxed at 300 K for 0.1 ns, and the results are shown in Supplementary Figure 8c, d. Then, a strain ε along [001] direction was loaded with the rate of 0.01/ps to make the edge dislocations glide. The dynamic glide process is shown in the supporting materials. The oxygen vacancy is stable and does not move before interacting with the dislocation core. Supplementary Figures 8e and 8f show the process that the dislocation moves across the oxygen vacancy. When V_O is close to the center of dislocation core, the oxygen atoms that are collinear with V_O along the glide direction move fast. Accordingly, the migration velocity of dislocation line becomes higher near V_O, creating a kink at this position. The results indicate that oxygen vacancies may help the formation of kinks in $SrTiO_3$. However, it's hard to record the migration process of the kink.

In order to analyze the effect of oxygen vacancy on dislocation motion, we recorded the migration process of dislocation line on as shown in Supplementary Figure

8g. A single oxygen vacancy has no influence on the glide of dislocation line, but a cluster of three oxygen vacancies shows a pinning effect on the moving dislocation, which effectively impedes the motion of dislocation line. The difference may be ascribed to the fact that the size of lattice strain near an oxygen vacancy is too small to affect the motion of dislocation line. In conclusion, a single oxygen vacancy may help with kink formation, but has little effect on dislocation motion. The cluster of oxygen vacancies has a pinning effect on dislocation motion.

Supplementary References

[1] Thomas et al., Nucl. Instrum. Methods Phys. Res. Sect. B Beam Interact. Mater. At. 2005, 239: 191-201.

[2] Yildiz et al., J. Am. Chem. Soc. 2015, 137: 4735-4748

[3] D. Brunner et al., Acta Mater. 2006, 54: 4999-5011.

[4] P. Carrez et al., Scripta Mater., 2010, 63: 434-437.

[5] M. Tang et al., Acta Mater., 1998, 46: 3221.

[6] D. Brunner et al., Phys. Stat. Sol. (a), 1991, 124: 155.

[7] Yang et al., J. Am. Ceram. Soc., 2011, 94: 3104-3111.

[8] S. Stich et al., J. Am. Ceram. Soc. 2022, 105: 1318-1329.

[9] R. A. De Souza, Adv. Funct. Mater. 2015, 25: 6326-42.
